# Performance of expanded non-invasive prenatal testing for fetal aneuploidies and copy number variations: A prospective study from a single center in Jiangxi province, China

**DOI:** 10.3389/fgene.2022.1073851

**Published:** 2023-01-13

**Authors:** Yongyi Zou, Chuanxin Feng, Jiawei Qin, Xinrong Wang, Tingting Huang, Yan Yang, Kang Xie, Huizhen Yuan, Shuhui Huang, Bicheng Yang, Wan Lu, Yanqiu Liu

**Affiliations:** ^1^ Department of Medical Genetics, Jiangxi Maternal and Child Health Hospital, Nanchang, Jiangxi, China; ^2^ Jiangxi Key Laboratory of Birth Defect Prevention and Control, Jiangxi Maternal and Child Health Hospital, Nanchang, Jiangxi, China

**Keywords:** expanded non-invasive prenatal testing (NIPT-plus), chromosome aneuploidies, copy number variations, positive predictive value, Jiangxi province

## Abstract

To evaluate the performance of expanded non-invasive prenatal testing (expanded noninvasive prenatal testing, NIPT-Plus) in screening for fetal chromosomal abnormalities includes aneuploidies and copy number variations, a total of 23,116 pregnant women with a singleton pregnancy were recruited for NIPT-Plus. Screening positive results were verified by karyotype analysis and chromosomal microarray analysis after amniocentesis. A total of 264 pregnancies (1.14%) were positive results as predicted by NIPT-Plus, including 233 aneuploidies and 31 copy number variations. Following genetic counseling, 233 (88.26%) pregnant women underwent invasive prenatal diagnosis and 136 were verified as true positives, comprising 72 common trisomies (T21, T18, T13), 47 sex chromosomal abnormalities two rare autosomal aneuploidies (RATs) and 15 copy number variations The positive predictive value for common trisomies, SCAs, RATs and CNVs were 68.57%, 68.12%, 6.67% and 51.72%, respectively. Pregnant women with screen-positive results for common trisomies have higher rates of invasive prenatal diagnosis and pregnancy termination than those with positive results for SCAs, RATs, and CNVs. NIPT-Plus showed a good performance in detecting common trisomies, SCAs and also contributed to detecting pathogenic CNVs, but higher accuracy was required in the detection of RATs. In summary, this study provides a reference for the clinical application of NIPT-Plus for screening fetal chromosomal abnormalities in this region. Therefore, we suggest that NIPT-Plus could be widely used in clinical screening for fetal chromosomal abnormalities in combination with prenatal diagnosis and genetic counseling.

## Introduction

Chromosomal abnormalities, which include aneuploidies and copy number variations, can result in a spectrum of diseases ranging from pregnancy loss to adults with psychiatric disorder (Yilmaz et al., 2017; Dahdouh and Kutteh, 2021). Most aneuploid embryos undergo embryonic death and spontaneous abortion in early development due to severe developmental defects. However, some trisomic aneuploid embryos can continue to develop and cause birth defects, such as trisomy 21, trisomy 18, trisomy 13 and SCAs(Hassold et al., 2007). In newborns, the prevalence of aneuploidy is approximately 0.3%, with the most common abnormalities being trisomy 21 and sex chromosome trisomy (Mikwar et al., 2020). Copy number variants are always associated with human genetic diseases and syndromes, and these changes from the genome can lead to gene dosage effects, gene breaking that affect gene expression levels and phenotypes (Grati et al., 2015). Microdeletion and microduplication syndromes (MMS) caused by pathogenic CNVs can occur in any pregnancy independent of maternal age (Chau et al., 2019). Clinically relevant CNVs have been identified in 6.0% of fetuses with structural anomalies on ultrasonography, and in 1.7% of pregnancies with standard indications for prenatal diagnosis (such as advanced maternal age and positive screening results) in previous studies (Wapner et al., 2012). Overall, chromosomal abnormalities occur in approximately one in 150 live births and are the main cause of congenital malformations and even death in infants and childhood (Carlson and Vora, 2017). Karyotyping and/or chromosomal microarray analysis after amniocentesis or chorionic villous sampling (CVS) can effectively detect fetal chromosomal abnormalities. However, the limitations of these techniques are their invasiveness and procedure-related miscarriage with a low but non-negligible risk (Alfirevic et al., 2017).

The discovery of cell-free fetal DNA (cffDNA) in maternal plasma and the rapid development of high-throughput sequencing technologies have enabled the non-invasive detection of fetal chromosomal abnormalities (Chitty and Lo, 2015), known as non-invasive prenatal testing (NIPT). NIPT has been widely used as a routine screening method for fetal chromosomal aneuploidy, and it shows high accuracy in detecting common trisomies. It was reported that the detection rate, specificity, and false positive rate of NIPT for common trisomies were 98.59%, 99.99%, and 0.02%, respectively (Yu et al., 2017). Numerous studies have shown that the PPV is in the range of 65%–94% for T21, 47%–85% for T18, 12%–62% for T13, and 45%–58% for SCAs(Quezada et al., 2015; Zhang et al., 2017; Zheng et al., 2020). The American College of Medical Genetics and Genomics (ACMG) recommended that NIPT could replace conventional screening for common trisomies in 2016 (Gregg et al., 2016).

In recent years, NIPT has expanded to detect rare autosomal aneuploidies (RATs) and copy number variations (CNVs) through deeper sequencing and higher level analyses. Several recent studies have demonstrated the possibility of expanded NIPT screening for fetal CNVs associated with MMS(Liang et al., 2019; Shi et al., 2021), including but not limited DiGeorge syndrome, Prader–Willi/Angleman syndrome, cri du chat syndrome, and 1p36 deletion syndrome. The performance of the expanded NIPT to detect MMS varied widely, with positive predictive values ranging from low (11%–18%) to moderate (29%–77%) (Shi et al., 2021). As a result, the accuracy of expanded NIPT in detecting chromosomal abnormalities other than common trisomies remains questionable (Christiaens et al., 2021). In addition, most studies of expanded NIPT originate from multiple centers in different regions. There are differences in the performance of expanded NIPT screening for chromosomal abnormalities reported by different centers. Therefore, large-scale prospective studies from a single center are necessary to guide the application of extended NIPT in local clinical practice.

In this study, we recruited a total of 23,116 singleton pregnancies who underwent expanded NIPT at a single local center to evaluate the performance of expanded NIPT in screening for aneuploidies and pathogenic CNVs. Our data have reference significance for the local clinical application of extended NIPT for screening fetal chromosomal abnormalities.

## Materials and methods

### Participant recruitment

This study recruited 23,116 singleton pregnant women consecutively from October 2019 to December 2021 at the Medical Genetics Center of Jiangxi Maternal and Child Health Hospital. Base on different clinical indications, we present four common risk factors as follows: ultrasound soft index abnormalities (echocardiographic lesions, choroid plexus cysts, single umbilical artery, *etc.*), positive serum screening (high or intermediate risks for serum screening), advanced maternal age (age ≥35 years), adverse reproductive history (previous adverse pregnancy outcomes). Pregnant women with one of the above risk factors are classified as a high-risk group, while those with none of the above risk factors are defined as a low-risk group. Written informed consent and pretest genetic counseling were provided to all pregnant women, including test objectives, significance, and limitations.

### Sample preparation and sequencing

Approximately 5 ml peripheral blood was collected in a cfDNA storage tube (Streck Cell Free DNA BCT, United States) from pregnant woman, and plasma DNA was extracted within 96 h of collection. Plasma was separated from blood samples using a two-step centrifugation protocol (Dan et al., 2012). The collected blood samples were first centrifuged at 1,600 g and 4°C for 10 min. The supernatant was then centrifuged at 16,000 g and 4°C for 10 min to remove residual blood cells. Cell-free DNA was extracted from plasma using a cfDNA isolation kit (BGI Biotechnology Co., Ltd., Wuhan) according to the reagent instructions. The extracted cfDNA was end repaired in a 10 ul end repair reaction mixture at 37°C for 10 min and 65°C for 15 min. Next, 30ul of the adapter ligation reaction mixture was added and incubated at 23°C for 20 min. Then 29ul PCR reaction mixture was added and PCR amplification was performed at 98°C 2 min, 12cycles (98°C 15 s, 56°C 15 s, 72°C 30 s) and 72°C 5 min to obtain cfDNA library. After quantifying with a QubitTM fluorometer (Thermo Fisher Scientific, United States), each cfDNA library was pooled at about 168ng. Libraries were tag sequenced with 45-cycle single-end sequencing on BGISEQ-500 platforms (BGI, China) to generate approximately 20 M Reads. Sequencing data were processed by a Non-invasive Fetal Trisomy (NIFTY) system as previously described (Jiang et al., 2012). Chromosome aneuploidies risk was evaluated using a ratio Z-score (|Z| ≥ 3 indicates a high risk of chromosomal aneuploidies, and |Z| < 3 indicates a low risk). Furthermore, algorithm based on hidden Markov models (HMM) was used to detect CNVs (Liang et al., 2019).

### Prenatal diagnosis and pregnancy follow-up

Genetic counseling and prenatal diagnostic testing would be offered to women receiving a high-risk screening result. T21, T18, and T13 trisomies and sex chromosome aneuploidies were confirmed using invasive amniocentesis followed by karyotyping, while CNVs high-risk results were confirmed by chromosomal microarray analysis (CMA). We then collected their invasive prenatal diagnostic results and pregnancy outcomes. Pregnant women with low-risk screening results are recommended for routine prenatal care. The postnatal follow-up was conducted by phone interview at 3 months after the expected date of delivery based on the guidelines of the National Health Commission of the People’s Republic of China (2016).

### Statistical analysis

Based on the results of expanded NIPT and invasive prenatal diagnosis, the sensitivity, specificity, positive predictive value (PPV), and negative predictive value (NPV) were calculated. Sensitivity = TP/(TP + FN), specificity = TN/(FP + TN), FNR = FN/all cases, FPR = FP/all cases, PPV = TP/(TP + FP), and negative pre-dictive value (NPV) = TN/(FN + TN). TP, FN, TN, and FP stand for true positive, false negative, true negative, and false positive, respectively. Cases without a confirmatory diagnosis or lost to follow-up were excluded from the study. All data analyses were performed using Statistical Product and Service Solutions (SPSS) software version 26.0. The Chi-square test were used to estimate the statistical significance between two categorical variables. *p* < 0.05 was considered statistically significant.

## Results

### Patient characteristics

**FIGURE 1:**
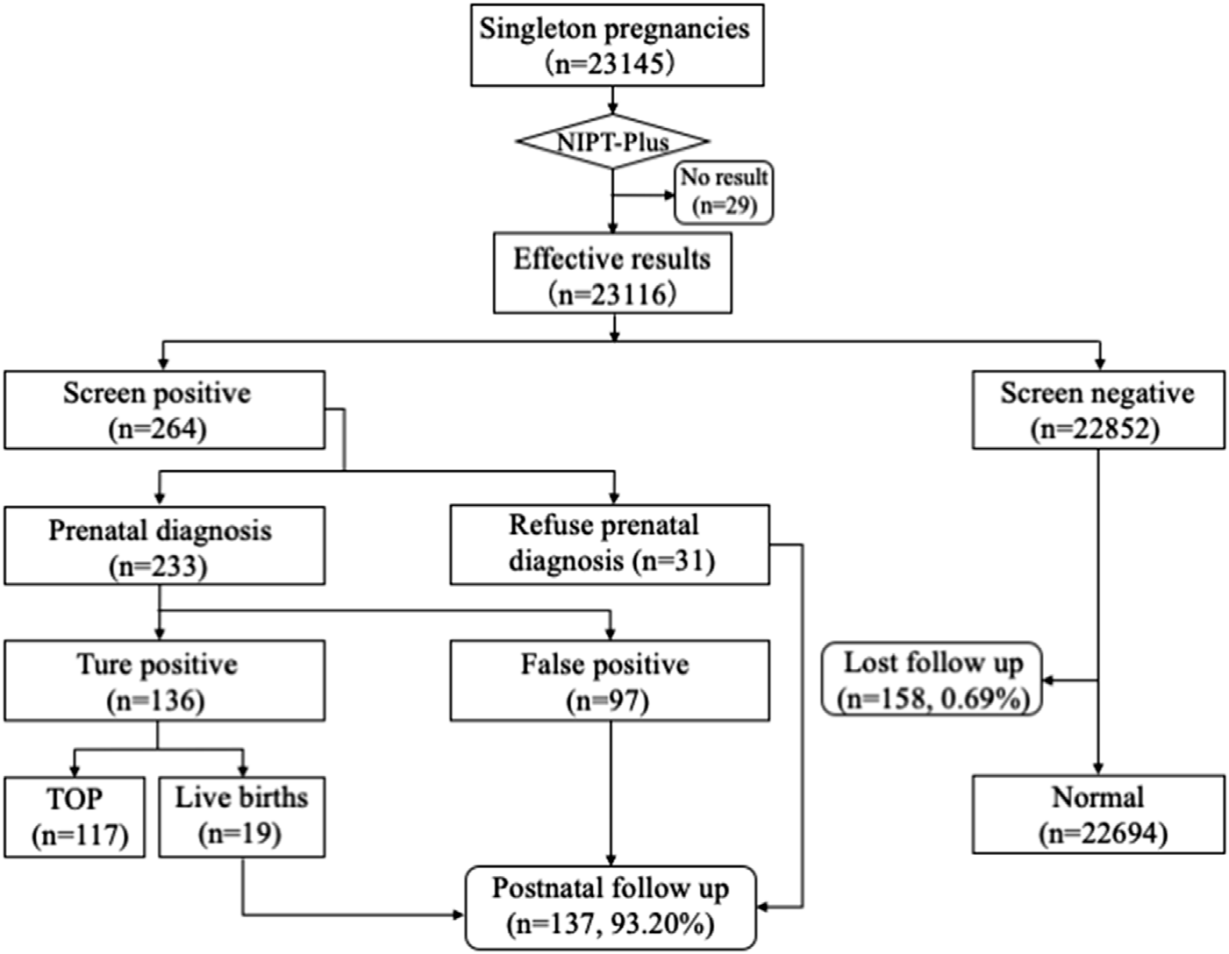
Flowchart of NIPT-Plus results and outcomes of pregnant women. TOP, termination of pregnancy.

A total of 23,145 pregnant women with singleton pregnancy were offered NIPT-Plus at a local center during the study period. 144 cases required repeat blood sampling due to poor quality of blood or low fetal DNA fraction (<3.5%), and valid results were obtained in 115 cases. The remaining 29 cases (0.13%) still had a low fetal fraction (<3.5%) and were excluded from this study (Figure 1). We followed up these 29 pregnant women, of whom 21 gave birth to normal children and eight were lost to follow-up.

Ultimately, a total of 23,116 samples were included in this study. The maternal age ranged from 14 to 54 years with a mean age of 29.6 years (SD, 4.9), and the gestational age ranged from 12 to 33 weeks with a mean gestational age of 15.5 weeks (SD, 2.7). The majority (83.67%) of the women were at 12–17^+6^ weeks when NIPT-Plus was performed. Of these 23,116 pregnant women, 8,395 pregnancies demonstrated single or multiple indications, including advanced maternal age, positive serum screening, ultrasound soft index abnormalities and adverse reproductive history (Table 1).

**TABLE 1:** Demographics and clinical characteristics of pregnancies.

Characteristic	Number	Percent (100%)
Maternal age (years)		
<25	3,549	15.35
25–29	7,988	34.56
30–34	7,664	33.16
35–39	3,350	14.49
≥40	565	2.44
GA at sampling (weeks)		
12–14^+6^	11,105	48.04
15–17^+6^	8,236	35.63
18–20^+6^	2,565	11.10
21–23^+6^	899	3.89
≥24	311	1.35
Clinical indications		
AMA	3,913	16.93
Positive serum screening	3,890	16.83
Ultrasound soft index abnormalities	517	2.24
Adverse reproductive history	528	2.28

GA, gestational age; AMA, advanced maternal age (≥35 years).

### Performance of NIPT-Plus for screening common trisomies and SCAs

Among 23,116 pregnant women screened by NIPT-Plus, there were 264 (1.14%) cases reported to be positive (high-risk). There were 109 positive cases for common trisomies (60 cases of T21, 36 cases of T18 and 13 cases of T13) and 84 positive cases for SCAs (32, 12, 28 and 12 cases of 45,X, 47,XXX, 47, XXY and 47,XYY, respectively). For common trisomies, 105 cases received invasive prenatal diagnosis, and 50 T21, 19 T18 and three T13 were confirmed. Therefore, the positive predictive value (PPV) for screening T21, T18 and T13 were 86.21%, 55.88% and 23.08%, respectively. All low-risk cases of NIPT-Plus were verified to be true negative, except 158 cases, which had been lost to follow-up. Thus, the compound PPV, NPV, sensitivity and specificity for common trisomies were 68.57%, 100%, 100% and 99.86%, respectively. Meanwhile, 69 cases of SCAs received prenatal diagnosis, and 47 true positive and 22 false positive SCAs cases were confirmed, resulting in a compound PPV of 68.12%. Among four categories of SCAs, the PPV of 47, XXX (88.89%), 47, XXY (99.15%) and 47, XYY (72.73%) were higher than that of 45, X (26.09%) (Table 2). Overall, NIPT-Plus demonstrated high sensitivity and specificity for the detection of common trisomies and SCAs.

**TABLE 2:** The performance of NIPT-Plus in screening for chromosome aneuploidy and copy number variations.

Chromosome abnormalities	TP	FP	PPV	Sensitivity (95% CI)	TN	FN	NPV (%)	Specificity (95% CI)
Common trisomies	72	33	68.57%	100% (93.69%–100%)	22,822	0	100	99.86% (99.79%–99.90%)
T21	50	8	86.21	100% (91.11%–100%)	22,869	0	100	99.97% (99.93%–99.98%)
T18	19	15	55.88	100% (79.08%–100%)	22,893	0	100	99.93% (99.89%–99.96%)
T13	3	10	23.08	100% (30.99%–100%)	22,914	0	100	99.96% (99.92%–99.98%)
SCAs	47	22	68.12	100% (90.59%–100%)	22,858	0	100	99.90% (99.85%–99.94%)
RATs	2	28	6.67	100% (19.79%–100%)	22,897	0	100	99.88% (99.82%–99.92%)
CNVs	15	14	51.72	100% (74.65%–100%)	22,898	0	100	99.94% (99.89%–99.97%)
<10Mb	8	8	50.00	100% (59.77%–100%)	22,911	0	100	99.86% (99.93%–99.98%)
≥10Mb	7	6	53.85	100% (56.09%–100%)	22,914	0	100	99.97% (99.94%–99.99%)

PPV, positive predictive; NPV, negative predictive value; TP, true positive; FP: false positive; TN, true negative; FN: false negative; SCAs, sex chromosome abnormalities; RATs, rare autosomal aneuploidies; CNVs, copy number variations.

### Performance of NIPT-Plus for screening RATs and CNVs

Among the 23,116 cases, 40 positive cases for RATs were reported. Confirmatory testing was performed in 30 of the 40 RATs cases. Two cases confirmed to be mosaic trisomy (mosaic T9 and mosaic T3), and the other 28 RATs cases had normal karyotype. Therefore, the combined PPV for RATs was as low as 6.67%. Thirty-one cases with positive CNVs results were reported, of which 29 cases underwent amniocentesis and CMA analysis. Fifteen cases had consistent results with NIPT-Plus, while the other 14 were discordant. Thus, the total PPV for CNVs was 51.72%. CNVs were categorized into two groups according to length (CNV<10Mb, and CNVs ≥10 Mb), and the PPV was calculated separately. There was no significant difference in PPV between CNV< 10Mb and CNV ≥10Mb positive cases (50.00% vs 53.85%, *p* = 0.837). The 15 true positive CNVs detected by NIPT-Plus were summarized in Table 3. 22q11 deletion syndrome (DiGeorge syndrome) was the most common (n = 3), followed by cri du chat syndrome (n = 2).

**TABLE 3:** Positive CNVs detected by NIPT-PLus.

Case	Age		NIPT-plus		Ture or false positive	Pathogenicity^a^		Pregnancy outcome
	Maternal (years)	Fetal (weeks)	Screening results	CNV size (Mb)	Confirmed by CMA	Syndrome	Classification	
1	37	13	Dup 1q41-q44	25.28	Ture	NA	P	TOP
2	33	18	Dup 4p14-p16.3	37.39	Ture	NA	P	TOP
3	34	13^+1^	Del 2q37.1q37.3	8.76	Ture	2q37 microdeletion syndrome	P	TOP
4	34	15^+1^	Del 5p15.33-p15.1	16.13	Ture	Cri du Chat syndrome	P	TOP
5	28	18^+6^	Del 5p15.33-p15.2	14.52	Ture	Cri du Chat syndrome	P	TOP
6	29	13^+2^	Del 10p13-p14	6.56	Ture	DiGeorge syndrome 2 (DGS2)	P	TOP
7	30	16^+3^	Dup 12p12.2-p13.33	20.81	Ture	NA	LP	TOP
8	27	19^+1^	Del 13q31.1-q31.3	9.34	Ture	NA	LP	TOP
9	36	16^+1^	Dup 13q31.2-q34	24.88	Ture	NA	P	TOP
10	28	26	Del 15q11.2-q13.1	5.22	Ture	Prader-Willi/Angelman syndrome	P	TOP
11	28	13	Del 22q11.21	1.44	Ture	22q11 deletion syndrome	P	TOP
12	26	14	Del 22q11.21	3.37	Ture	22q11 deletion syndrome	P	Birth
13	25	12	Del 22q11.21-q11.23	8.00	Ture	22q11 deletion syndrome	P	TOP
14	33	12^+3^	Del Xq21.1-q21.33	22.30	Ture	Xq21 deletion syndrome	P	TOP
15	28	18^+1^	Del Xq28	5.54	Ture	Chromosome Xq28 deletion syndrome	P	Birth
16	28	25	Dup 22q11.21-q12.1	8.19	False			Birth
17	34	17	Del 7q21.13-q31.32	32.17	False			Birth
18	37	13^+4^	Del 1p36.32	10.55	False			Birth
19	21	17^+1^	Del 8p23.1	3.66	False			Birth
20	29	15^+6^	Del Xq22.3	5.00	False			Birth
21	32	16^+5^	Del 5p15.33-p15.2	10.53	False			Birth
22	29	18	Del 17p13.3	3.30	False			Birth
23	23	16	Del 8p23.3-p12	32.80	NA			TOP
24	28	17	Del 15q11.2-q13.1	5.69	False			Birth
25	31	14^+1^	Dup Xp22.33-q11.2	61.32	False			Abortion
26	44	16^+5^	Del 15q11.2q13.1	3.01	False			Birth
27	32	19^+3^	Del 5p15.33-p15.32	3.00	False			Birth
28	37	18	Del 1p35.1-p36.33	32.41	NA			Birth
29	40	12 + 6	Del 1p31.1-p32.1	10.74	False			Birth
30	24	13^+3^	Dup 8p22-p23.3	14.88	False			Birth
31	29	14^+2^	Del 7q21.3-q31.33	32.67	False			Birth

Del, deletion; Dup, duplication; TOP, termination of pregnancy; P, pathogenic; LP, likely pathogenic.

^a^ Data from OMIM database (https://omim.org/) or.

DECIPHER database (https://decipher.sanger.ac.uk/); NA, not applicable.

### Performance of NIPT-Plus screening for chromosomal abnormalities in high- and low-risk groups

Based on different clinical indications, 8,395 (36.3%) pregnant women were classified as high-risk groups and 14,721 (63.7%) as low-risk groups. The positive rate and PPV of T21, T18, T13, SCAs, RATs and CNVs in the two groups are shown in Table 4. For T21, whose higher disease prevalence is known to associated with advanced maternal age, both positive rate and PPV were significantly higher in the high-risk groups than in the low-risk groups (PR 0.43% vs 0.16%, PPV 94.29% vs 73.91%, *p* < 0.05). However, there was no significant difference between the two groups in PPV for T18, T13, SCAs, RATs, and CNVs (T18 58.33% vs. 54.55%, T13 25.00% vs. 22.22%, SCAs 70.37% vs 66.67%, RTAs 16.67% vs. 4.17%, CNVs 50.00% vs 52.63%, *p* > 0.05). At the same time, the positive rates of these chromosomal abnormalities also showed similar characteristics between the two groups.

**TABLE 4:** Performance comparison of NIPT-Plus between high-risk and low-risk groups.

Chromosome abnormalities	High-risk group (*n* = 8,395)			Low-risk group (n = 14,721)		
	Positive	PR (%)	PPV (%)	Positive	PR (%)	PPV (%)
T21	36	0.43*	94.29*	24	0.16	73.91
T18	13	0.15	58.33	23	0.16	54.55
T13	4	0.05	25.00	9	0.06	22.22
SCAs	33	0.39	70.37	51	0.35	66.67
RATs	12	0.14	16.67	28	0.19	4.17
CNVs	11	0.13	50.00	20	0.14	52.63

PR, positive rate; PPV, positive predictive; *< 0.05, Chi-square test.

### Pregnancy outcome

All the 264 cases of NIPT-Plus positive results were followed up to pregnancy outcomes (Table 5). A total of 72 positive results were confirmed as common trisomies (50 T21, 19 T18, and 3 T13), all of whom opted for termination of pregnancy (TOP). Thirty-one women carrying fetuses with SCAs terminated their pregnancy, with higher rates of termination in cases of 45,X (66.7%) and 47,XXY (88%) than in cases of 47, XXX (37.5%) and 47, XYY (25%). In addition, the termination rates for cases confirmed as RATs and CNVs were 50% (1/2) and 86.7% (13/15), respectively. For 31 cases with a NIPT-Plus positive result who refused prenatal diagnosis, 10 (32.3%) opted for termination and 21 (67.7%) continued the pregnancy. A total of 100% (4/4) of unconfirmed common trisomy positive cases terminated their pregnancy, which was higher than unverified positive cases for SCAs (20%, 3/15), RATs (20%, 2/10) and CNVs (50%, 1/2).

**TABLE 5:** Pregnancy outcome of confirmatory abnormal fetuses and positive screening cases without prenatal diagnosis.

Chromosome abnormlities	Ture positive (karyotyping or CMA)	Pregnancy outcome, *n* (%)		Refuse prenatal diagnosis	Pregnancy outcome, *n* (%)	
		TOP	Live birth		TOP	Live birth
T21	50	50 (100)	0	2	2 (100)	0
T18	19	19 (100)	0	2	2 (100)	0
T13	3	3 (100)	0	—	—	—
45,X	6	4 (66.7)	2 (33.3)	9	1 (11.1)	8 (88.9)
47,XXX	8	3 (37.5)	5 (62.5)	3	0	3 (100)
47,XXY	25	22 (88)	3 (12)	2	2 (100)	0
47,XYY	8	2 (25)	6 (75)	1	0	1 (100)
RATs	2	1 (50)	1 (50)	10	2 (20)	8 (80)
CNVs	15	13 (86.7)	2 (13.3)	2	1 (50)	1 (50)

CMA, chromosomal microarray analysis; TOP, termination of pregnancy.

## Discussion

NIPT for prenatal screening of T21, T18, and T13 has been widely accepted by clinicians and patients in recent years. However, whether this approach can be extended to detect other chromosomal abnormalities remains controversial (Evans et al., 2016; Chitty et al., 2018). Several recent studies have demonstrated the application of expanded NIPT (NIPT-Plus) in screening for multiple chromosomal abnormalities, including SCAs, RATs, and CNVs. However, the PPV varies widely across studies, ranging from 33% to 59% for SCAs, 5%–29% for RATs, and 29%–61% for CNVs(Fiorentino et al., 2017; Chen et al., 2019; 2021; Ge et al., 2021). In present study, a cohort of 23,116 singleton pregnancies from a single center were enrolled to investigate the performance of NIPT-Plus. Our study showed that NIPT-Plus had a comparable PPV for T21 (86.21%) and T18 (55.88%) but a relatively low PPV for T13 (23.08%) compared to previous studies (Chen et al., 2022). The low PPV for T13 may be related to the small number of T13 positive cases in this study, which would increase the fluctuation of PPV. Another possible reason could be related to the size of chromosome 13 or the GC ratio on chromosome 13 (Hu et al., 2019). Furthermore, our data suggested that the sensitivity and specificity of NIPT-Plus for screening common trisomies were comparable to standard NIPT (Lu, 2020). In our study, the combined PPV for SCAs was 61.82%, which was similar to that reported by expanded NIPT (Ge et al., 2021). Overall, the PPV for XXX, XXY, and XYY were 88.89%, 96.15%, and 72.73%, respectively, which was obviously higher than the 26.09% of monosomy X. The poor accuracy of screening for monosomy X may be due to confined placental mosaicism, a vanishing twin affected by monosomy X or maternal monosomy X mosaicism (Zhao et al., 2022).

In our study, we also explored the applicability of NIPT-Plus in screening for other chromosomal abnormalities, such as fetal RATs and CNVs. We screened 40 positive cases for RATs with a low PPV of 6.67%, which was similar to that reported in a recent study (Ge et al., 2021). Despite the lack of investigation into the cause of false positive RATs, fetoplacental mosaicism has been identified as a potential explanation in previous case studies (Grati et al., 2014). Since most of RATs belong to non-viable trisomy and spontaneous abortion occurs in the first trimester, it is recommended to conduct genetic counseling and prenatal diagnosis (FISH or CMA) based on the results of NIPT-Plus and ultrasonography to confirm the presence of chromosomal trisomy mosaicism in the fetus. We also evaluated the efficiency of NIPT-Plus to screen for CNVs, with a total of 31 positive cases detected and 15 confirmed as true-positive cases, resulting in a PPV of 51.72%. The most common of the 15 true positive cases identified by CMA was DiGeorge syndrome, followed by cri du chat syndrome. Meanwhile, the PPV for DiGeorge syndrome and cri du chat syndrome was also relatively high in this study, which was consistent with Liang’s study (Liang et al., 2019). In addition, there was no significant difference in PPV for CNVs <10 Mb and CNVs ≥10 Mb (50.00% vs 53.85%, *p* = 0.837) in present study. This indicated the effectiveness of NIPT-Plus and validated algorithms for identifying CNVs, even for small CNVs.

The American College of Obstetricians and Gynecologists (ACOG) recommends screening for aneuploidy in all pregnancies (American College of Obstetricians and Gynecologists’ Committee on Practice Bulletins—Obstetrics, 2020). Therefore, it is necessary to evaluate the performance of NIPT plus for screening chromosomal abnormalities including aneuploidy and CNV in pregnancies with different risk factors. According to our results, 8,395 (36.32%) cases with high-risk factors chose NIPT-Plus screening, from which, a total of 109 positive cases were screened. Meanwhile, 155 positive cases were screened in 14,721 (63.7%) pregnant women without any risk factors. We further analyzed the performance of NIPT-Plus in the high-risk and low-risk groups. The results showed that the positive rate and PPV for T21 in the high-risk group were significantly higher than those in the low-risk group. However, the positive rate and PPV for other chromosomal abnormalities were not significantly different. Thus, it is suggested that NIPT-Plus could be widely used in all pregnant women as a routine prenatal screening.

Among the positive cases screened, 31 cases (11.74%) refused prenatal diagnosis, of which 10 cases chose to terminate pregnancy and 21 cases continued pregnancy. Compared with RATs and CNVs, pregnant women with positive results of common trisomies were more likely to choose for prenatal diagnosis. Furthermore, because of the severity of the three diseases, all cases diagnosed as common trisomy choose to terminate pregnancy, which was similar to Zhou’s study (Zhou et al., 2019). Pregnancy termination was chosen in 66% (31/47) of confirmed SCA cases, which was comparable to other studies in the same period (61.1%–81%) (Scibetta et al., 2017; Lai et al., 2021). The pregnancy termination rate was 37.5% (3/8) for 47, XXX syndrome cases and 25% (2/8) for 47, XYY syndrome cases, which was significantly lower than other sex chromosome syndromes. This suggests that there is a growing acceptance of children with 47, XXX and 47, XYY syndrome, whose IQs are mostly in the normal range despite occasional physical abnormalities observed (Berglund et al., 2019). However, 80% (12/15) of the positive cases of SCAs without prenatal diagnosis opted for continuing pregnancies, which was significantly higher than that with confirmation of SCAs. Prenatal genetic counseling and the cognition level of pregnant women may have contributed to these differences. We also noted that pregnant women with confirmed larger CNVs or CNVs associated with MMS chose to terminate their pregnancy. There was one case chose to continue the pregnancy after it was confirmed to be inherited from the parents, and succeeded in live birth. This suggests that the origin of CNVs must be identified if necessary, which is important for genetic counseling. In addition, we also followed up 21 cases of continued pregnancy 3 months after delivery. Among them, one case was preterm at 31 weeks and had difficulty feeding at birth but no other structural abnormalities were found. No obvious abnormality was found in the other 20 patients during follow-up. We will continue to follow up for 1 and 3 years after birth to track the development.

There were several limitations in this study that should be noted. Screening negative cases lost to follow-up and positive cases without prenatal diagnosis were excluded when assessing NIPT-Plus performance, which may have affected the accuracy of our results. In accordance with the National Health Commission guidelines of China, follow-up began 12 weeks after delivery. Although there were no further false-negative case reports, it is too early to judge the chromosomal abnormalities merely through our follow-up at 3 months after delivery, especially for SCAs and CNVs. Unlike common trisomies (T21/T18/T13) that show distinct physical signs of chromosomal disease at birth, symptoms of SCAs and pathogenic CNVs may not appear until childhood (Battaglia et al., 2013; Skuse et al., 2018). Thus, another limitation to this study is that longer follow-up will be necessary to accurately assess the performance of NIPS-Plus for detecting SCAs and pathogenic CNVs.

By increasing sequencing depth and optimizing algorithms, NIPT-Plus can not only be used in screening for common trisomies, but also for other chromosomal abnormalities, such as SCAs, RATs, and CNVs. Our data demonstrated that NIPS-Plus shows high performance in detecting common trisomes and SCAs and also contributed to detecting pathogenic CNVs. Moreover, NIPT-Plus showed comparable performance in low-risk populations as in high-risk populations. This study is the first large-scale NIPT-Plus analysis of pregnant women in Jiangxi, China, and provided sufficient data support for the clinical application of NIPT-Plus in this region. Although similar studies have been done in other regions of China, there are differences in the screening performance of NIPT-plus in different regions. The study reported in Fujian showed that the PPV of the T18 screening was as high as 78%, while the study in Guangxi was similar to ours with the PPV of only 52%. In addition, the PPV of SCAs screening in our study was 68%, which was higher than Fujian’s 59% and Guangxi’s 40% (Chen et al., 2021; Ge et al., 2021). These data have significant implications for genetic counseling by local physicians, reducing anxiety and potentially unnecessary pregnancy termination in some high-risk pregnant women. With the advancement of methodology and the accumulation of clinical experience, NIPT-Plus combined with conventional ultrasonography will be the most comprehensive and reliable method for detecting fetal chromosomal abnormalities.

## Data Availability

The original contributions presented in the study are publicly available. This data can be found in Figshare https://doi.org/10.6084/m9.figshare.21825066.v1.
