# Supplementary material for: Performance of expanded non-invasive prenatal testing for fetal aneuploidies and copy number variations: A prospective study from a single center in Jiangxi province, China
Source: Front Genet. 2023 Jan 13;13:1073851. doi: 10.3389/fgene.2022.1073851 (PMC9880269; doi:10.3389/fgene.2022.1073851)
Supplement: Supplementary file 1 [file Image1.pdf]

Supplyment figure1:

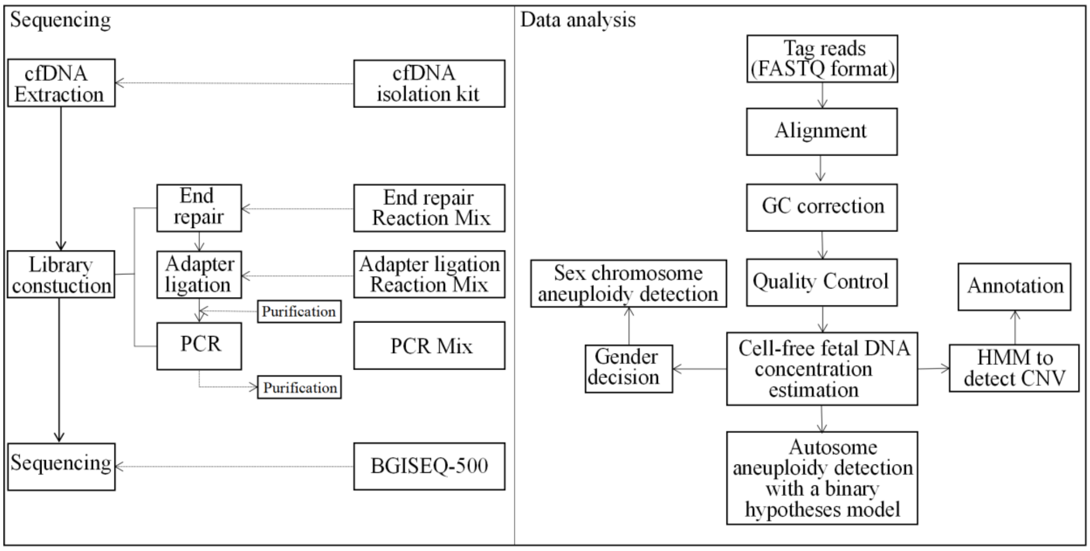

Supplyment Figure1: Screening pipeline for detection of chromosome aneuploidy and CNVs.

cfDNA, cell-free DNA; PCR, polymerase chain reaction; GC, guanine and cytosine content; CNV, copy-number variation
